# 12Cr2Mo1V Steel for Free-Forged Hydrogenation Reactor Shells: Defect Control, Microstructural Evolution, and Service Performance—A Review

**DOI:** 10.3390/ma19122464

**Published:** 2026-06-09

**Authors:** Haitao Wang, Guozheng Quan, Yichou Lin, Lin Gao, Yuqing Zhang, Xiao Liu, Haopeng Shi

**Affiliations:** 1 Chongqing Key Laboratory of Advanced Mold Intelligent Manufacturing, School of Materials Science and Engineering, Chongqing University, Chongqing 400044, China; 2 School of Materials Science and Engineering, Jiamusi University, Jiamusi 154007, China; 3 School of Mechanical and Electric Engineering, Sanming University, Sanming 365004, China; 4 Luoyang Zhongzhong Casting and Forging Co., Ltd., Luoyang 471033, China; 5 China National Erzhong Group Co., Ltd., Deyang 618000, China

**Keywords:** 12Cr2Mo1V steel, hydrogenation reactor shell, free forging, Cr-Mo-V steel, bainite, defect closure, carbide evolution, hydrogen service, thick-section forging

## Abstract

Hydrogenation reactor shells are safety-critical thick-section pressure-bearing components in petrochemical hydroprocessing equipment. Long-term exposure to elevated temperature, high pressure, and hydrogen-bearing media requires not only adequate strength, but also toughness, tempering stability, hydrogen-damage resistance, and through-thickness property uniformity. 12Cr2Mo1V steel, a Chinese Cr-Mo-V reactor steel closely related to vanadium-modified 2.25Cr-1Mo-0.25V steels, is widely used for large-shell forgings because its alloy design supports bainitic transformation, carbide stability, and elevated-temperature performance. This review critically synthesizes studies on 12Cr2Mo1V shell forgings, related Cr-Mo-V reactor steels, and heavy free-forged products. The discussion is organized around alloy design, ingot-derived defect inheritance, defect closure during free forging, bainite–grain–carbide evolution during forging and heat treatment, and the resulting strength, toughness, and hydrogen-service performance. Particular emphasis is placed on the process–defect–microstructure–property linkage in super-thick sections. The review shows that free forging is not merely a forming route, but a decisive metallurgical operation for densification, strain penetration, and precursor-structure conditioning. Future work should integrate casting, free forging, and heat treatment with multiscale characterization and data-enhanced predictive quality control. To further reduce descriptive comparison, this review summarizes standardized quantitative indicators for evaluating forging-route design, heat-treatment response, and prediction-method reliability.

## 1. Introduction

Hydrogenation reactors are among the most demanding pressure-bearing components used in hydroprocessing, hydrocracking, and residue-upgrading units. Their shells operate under the combined effects of elevated temperature, high internal pressure, and hydrogen-rich media; consequently, shell materials must be assessed in terms of strength, toughness, tempering stability, hydrogen-damage resistance, and long-term structural reliability rather than room-temperature strength alone [1,2,3,4,5,6,7]. For heavy-wall shell forgings, these requirements must be evaluated over the complete manufacturing and heat-treatment route.

**Figure 1:**
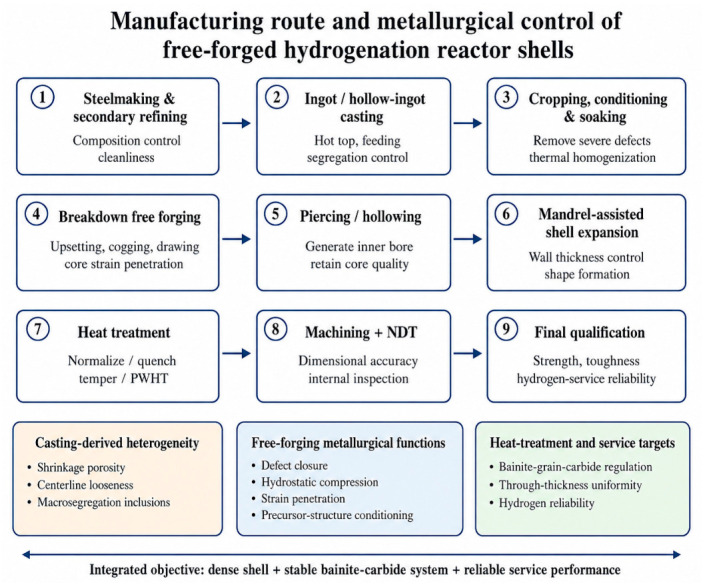
Corrected schematic manufacturing route for free-forged hydrogenation reactor shells, covering steelmaking and secondary refining, ingot/hollow-ingot casting, cropping and soaking, breakdown free forging, piercing or hollowing, mandrel-assisted shell expansion, heat treatment, machining and nondestructive testing, and final qualification. The schematic highlights the dual geometrical and metallurgical roles of free forging in defect closure, center consolidation, and precursor-structure conditioning.

As summarized in Figure 1, a typical route begins with steelmaking and secondary refining, followed by ingot or hollow-ingot casting, cropping and conditioning, high-temperature soaking, primary free forging for upsetting, cogging, drawing, and defect closure, piercing or hollowing, mandrel-assisted shell expansion, heat treatment, machining, nondestructive inspection, and final qualification. Free forging therefore acts simultaneously as a geometrical forming process and as a metallurgical operation for densification, strain penetration, and precursor-structure conditioning.

**Figure 2:**
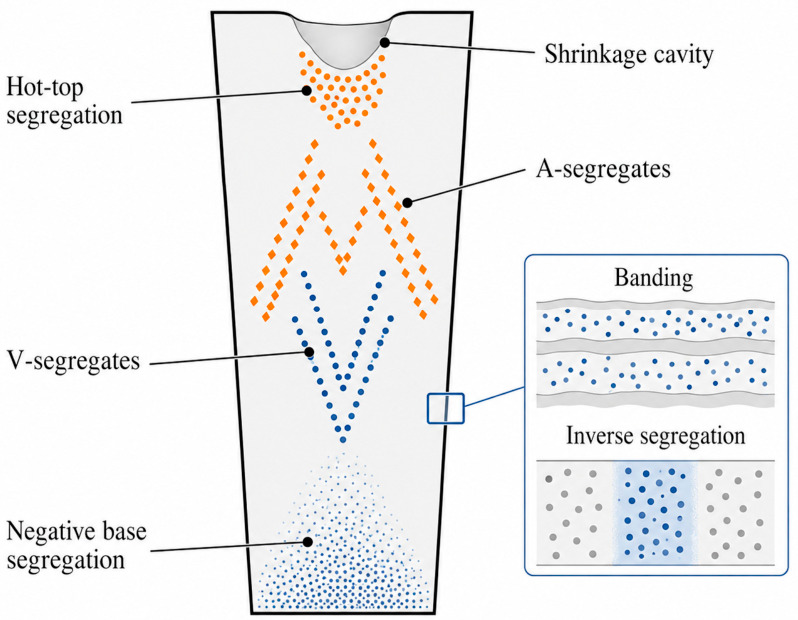
Schematic macrosegregation features in large steel ingots, including hot top segregation, A-segregates, V-segregates, negative base segregation, shrinkage cavity, banding, and inverse segregation.

The internal quality challenge originates at the ingot stage and is subsequently inherited by the forging route. Massive starting ingots may contain shrinkage porosity, centerline looseness, macrosegregation, and inclusion-enriched regions, all of which can reduce through-thickness property uniformity if they are not effectively controlled. As schematically shown in Figure 2, large steel ingots commonly exhibit A-segregation, V-segregation, negative base segregation, and shrinkage-related features, which together define the inherited-defect background of heavy shell forgings [8,9,10,11,12,13,14,15]. During subsequent forging and heat treatment, pronounced gradients in temperature, strain, and cooling rate may further amplify microstructural and property inhomogeneity. Free forging should therefore be treated not merely as a shaping operation but as a critical metallurgical stage for densification, defect mitigation, internal quality improvement, and precursor-structure conditioning in large hydrogenation reactor shell components [16,17].

Within this engineering context, vanadium-modified Cr-Mo steels have become important materials for modern hydrogen-service vessels because they provide a favorable balance of elevated-temperature strength, tempering resistance, and hydrogen-service performance compared with conventional 2.25Cr-1Mo steels. In Chinese engineering practice, hydrogenation reactor steels are often described as 12Cr2Mo1V or 12Cr2Mo1VR-type steels, whereas the international literature more commonly uses the designation 2.25Cr-1Mo-0.25V for closely related vanadium-modified Cr-Mo reactor steels. These two literature streams are complementary for a review centered on heavy-wall shell forgings because they address overlapping process–structure–property problems in low-carbon Cr-Mo-V steels for hydrogen-service equipment [1,2,3,4].

The suitability of this steel family is closely related to the microstructures produced during forging and heat treatment. Studies on large cast-forged Cr-Mo-V products and heavy-wall reactor steels show that bainitic morphology, grain-scale heterogeneity, carbide precipitation, and tempering response strongly affect strength, toughness, and hydrogen damage tolerance. Carbide stability is particularly important because it controls precipitation strengthening, tempering resistance, and the interaction between hydrogen and microstructural traps at elevated temperature [3,4,7,18,19].

**Figure 3:**
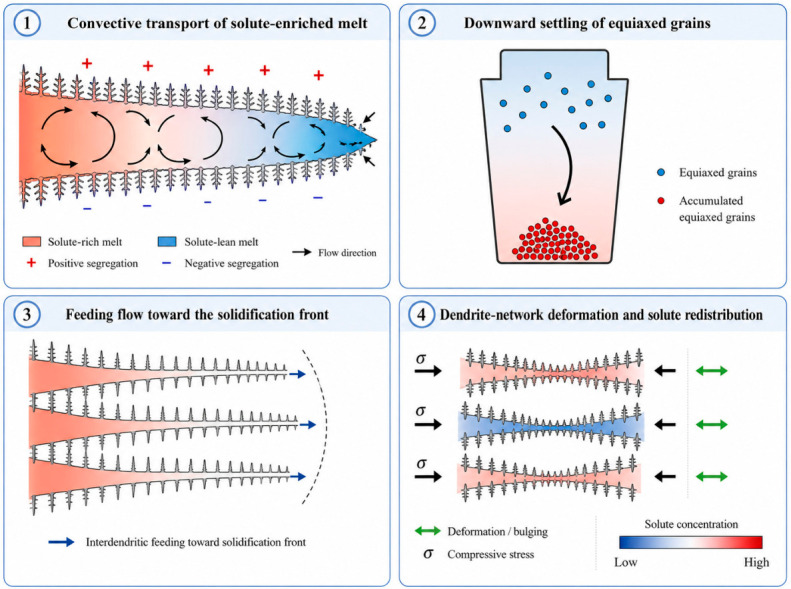
Main formation mechanisms of macrosegregation in large steel ingots, including solute-enriched liquid flow, equiaxed-crystal sedimentation, shrinkage-induced feeding flow, and deformation of the dendritic network.

The manufacturing problem is inseparable from the material problem. For heavy hydrogenation reactor shell forgings, internal quality is controlled by the defect state inherited from the ingot and by its subsequent evolution during solidification, forging, and heat treatment. Studies on heavy-ingot forging and defect healing show that sufficient deformation can markedly reduce or close internal cavities, whereas inadequate reduction, insufficient compressive stress, or an unfavorable deformation path may leave residual heterogeneity in the interior. As illustrated in Figure 3, macrosegregation in steel ingots is associated with thermosolutal convection, equiaxed-grain motion, shrinkage feeding, and deformation of the solid network [8,9,10,11,12,13,14,15]. These mechanisms explain why large ingots enter the forging stage with chemical and structural nonuniformity, which then becomes the initial condition for later property gradients. Even after apparent void closure, local metallurgical weakness may persist unless deformation amount, strain path, strain rate, and thermal schedule are coordinated. Therefore, a review of hydrogenation reactor shell forgings should integrate alloy design, defect mitigation, microstructural evolution, and service performance into a unified material–manufacturing–performance framework [17,20,21].

**Figure 4:**
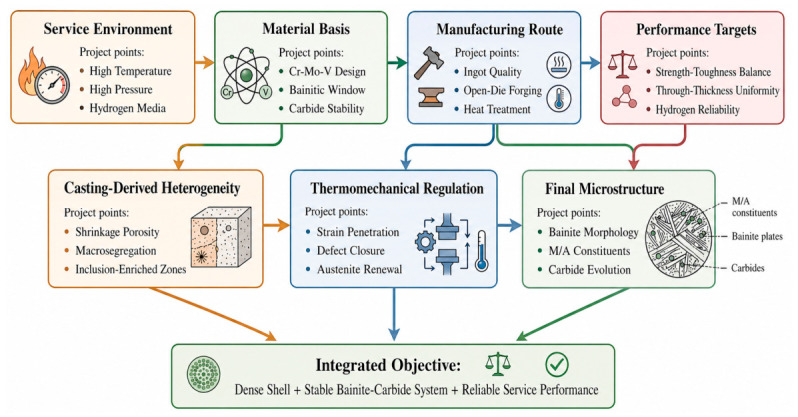
Review framework linking service environment, alloy design, ingot-derived heterogeneity, thermomechanical regulation, final microstructure, and service performance in 12Cr2Mo1V free-forged hydrogenation reactor shell forgings.

The present review focuses on 12Cr2Mo1V steel for hydrogenation reactor shell sections produced by free forging while drawing on the broader literature on metallurgically related 2.25Cr-1Mo-0.25V reactor steels and heavy free-forged products. It provides a critical synthesis of published results rather than new experimental data. The discussion is organized around five connected themes: material characteristics, defect mitigation during free forging, microstructural evolution during forging and heat treatment, mechanical and hydrogen-service performance, and future development directions. The objective is to clarify the process–defect–microstructure–property relationships that govern the internal quality and reliability of heavy-wall shell forgings. A concise literature map is provided in Table 1, and the overall review logic is summarized in Figure 4.

To define the review scope transparently, the literature survey combined targeted keyword searches with citation tracking in major publisher databases and academic search platforms. The principal search terms included 12Cr2Mo1V, 12Cr2Mo1VR, 2.25Cr-1Mo-0.25V, Cr-Mo-V reactor steel, hydrogenation reactor shell, heavy-wall forging, free forging/open-die forging, void closure, macrosegregation, bainite, retained austenite, carbide evolution, hydrogen embrittlement, high-temperature hydrogen attack, digital twin, and data-driven quality prediction. Foundational metallurgy and forging studies were retained when they clarified mechanisms transferable to heavy Cr-Mo-V forgings, whereas studies published from approximately 2000 to 2026 were prioritized for alloy-specific, processing-specific, and service-performance evidence.

The inclusion criteria emphasized peer-reviewed papers and technically traceable industrial studies that addressed at least one link in the process–defect–microstructure–property chain: alloy design, ingot-derived defects, deformation-path-dependent defect closure, bainite/M-A/retained-austenite/carbide evolution, through-thickness mechanical performance, hydrogen-service degradation, or predictive quality control. General papers on bainite, recrystallization, hot working, and hydrogen trapping were used only as mechanistic support. The available literature is strongest for Chinese engineering applications, international Cr-Mo-V reactor steels, European and East Asian forging/metallurgy studies, and open-access numerical modeling. Manufacturer-centered heavy-forging experience was considered where available, but was interpreted cautiously when processing data, sampling positions, or validation details were incomplete. Because the reviewed studies differ in component scale, sampling position, heat-treatment route, and validation depth, their evidence was interpreted using standardized comparison indicators, including core effective strain, void-closure evidence, surface–core property scatter, heat-treatment sensitivity, and model-validation error, rather than by qualitative claims alone.

## 2. Material Characteristics of 12Cr2Mo1V Steel

### 2.1. Chemical Composition and Alloy Design

12Cr2Mo1V belongs to the family of low-carbon Cr-Mo-V heat-resistant steels developed for heavy-wall pressure vessels operating under coupled high-temperature and hydrogen-bearing conditions. Its alloy design should not be interpreted as a conventional low-alloy strengthening strategy alone. Instead, it reflects a multi-objective metallurgical balance among hardenability, bainitic transformation control, tempering stability, precipitation strengthening, and resistance to hydrogen-related degradation. This design philosophy is particularly important for thick-section reactor shell forgings, in which thermal history, deformation degree, and cooling condition vary from the surface to the core, thereby imposing stringent requirements on through-thickness microstructural stability and property consistency. Studies on vanadium-modified 2.25Cr-1Mo-type reactor steels show that such alloys are intended to maintain a predominantly bainitic microstructure over a relatively wide cooling window while retaining tempering resistance and elevated-temperature strength for long-term service [4,5,6,8,18,19,22,23,24,25].

From the compositional point of view, carbon provides the basic strengthening potential and strongly affects transformation behavior, carbide precipitation, and toughness. Chromium and molybdenum mainly enhance hardenability, improve elevated-temperature strength, retard temper softening, and promote the formation of stable alloy carbides. Among the alloying elements, vanadium plays the most distinctive role in this steel family. It contributes to the precipitation of fine V-rich carbides or carbonitrides, improves tempering resistance, and helps stabilize the bainitic substructure during prolonged thermal exposure. In hydrogen-service steels, the significance of vanadium is not limited to strengthening alone; it is also associated with carbide-state regulation and thus with hydrogen-trapping behavior, hydrogen damage resistance, and long-term microstructural stability. Therefore, the alloy design of 12Cr2Mo1V should be understood as a coordinated strategy for simultaneously controlling phase transformation, precipitation behavior, and service reliability, rather than as an isolated composition adjustment for higher strength [18,19,22,24,26,27,28].

The engineering significance of this design becomes clearer when vanadium-modified Cr-Mo steels are compared with conventional 2.25Cr-1Mo steels without V addition. The introduction of vanadium was intended to improve high-temperature strength, creep resistance, tempering stability, and hydrogen-service performance while maintaining acceptable toughness for thick-wall pressure-vessel applications. This is especially important for hydrogenation reactor shell forgings, because such components operate for long periods under severe thermo-mechanical and hydrogen exposure conditions, and their failure risk is highly sensitive to metallurgical instability or local inhomogeneity. In this sense, 12Cr2Mo1V is better regarded as a reactor steel specifically optimized for the coupled requirements of high-temperature load bearing, thick-section heat treatment, and hydrogen-bearing service, rather than as a simple high-strength derivative of ordinary Cr-Mo steel [3,4,18,19,23,24,25].

Another important feature of this alloy design is its close relation to the target microstructure of heavy-wall products. Available studies on 2.25Cr-1Mo-0.25V steels show that the alloy tends to remain fully or nearly fully bainitic across a broad range of cooling conditions, which is highly desirable for large pressure-vessel sections because bainitic microstructures generally provide a favorable balance between strength, toughness, and thermal stability. This transformation tendency explains why vanadium-modified Cr-Mo steels are particularly suitable for large reactor shell forgings, where excessively narrow transformation windows would otherwise amplify section-thickness effects and cause marked microstructural gradients. Thus, the composition design of 12Cr2Mo1V is intrinsically linked to its processing route and final service role: the alloy chemistry is selected not only to meet compositional specifications, but also to support thick-section bainitic transformation, stable carbide evolution, and reliable long-term performance [7,16,19,29].

### 2.2. Low-Carbon Bainitic Microstructure

**Figure 5:**
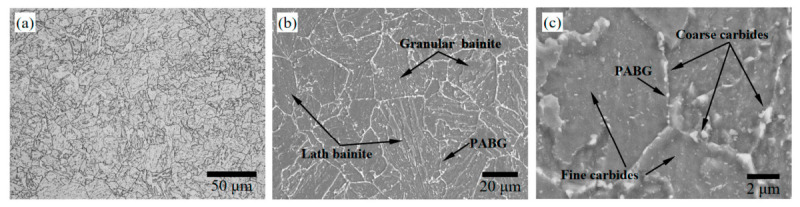
Representative as-received microstructure of 2.25Cr-1Mo-0.25V steel in the normalized-and-tempered condition. (**a**) Optical metallographic microstructure; (**b**) low-magnification SEM image showing granular bainite, lath bainite, and prior-austenite boundaries; (**c**) high-magnification SEM image showing fine and coarse carbides at prior-austenite boundaries [7].

For 12Cr2Mo1V heavy-wall shell forgings, the desired microstructure after forging and heat treatment is generally a low-carbon bainite-dominated structure rather than coarse ferrite–pearlite or fully martensitic products. From a metallurgical perspective, this target is well suited to thick-section reactor steels because bainitic microstructures usually provide a more favorable balance of strength, toughness, and thermal stability, while also offering a wider and more practical processing window for large cross-sections than fully martensitic structures. As shown in Figure 5, a representative normalized-and-tempered starting condition reported for the related 2.25Cr-1Mo-0.25V steel consists mainly of granular bainite and lath bainite, indicating that a bainite-dominated microstructural state is both realistic and technologically relevant for hydrogenation reactor applications [7]. Previous studies on 2.25Cr-1Mo-0.25V heavy-wall steels have likewise shown that the received or normalized-and-tempered condition is dominated by granular bainite together with lath bainite, further confirming that bainite is an appropriate microstructural target for hydrogenation reactor shell materials [7,16,19,29,30,31,32].

**Figure 6:**
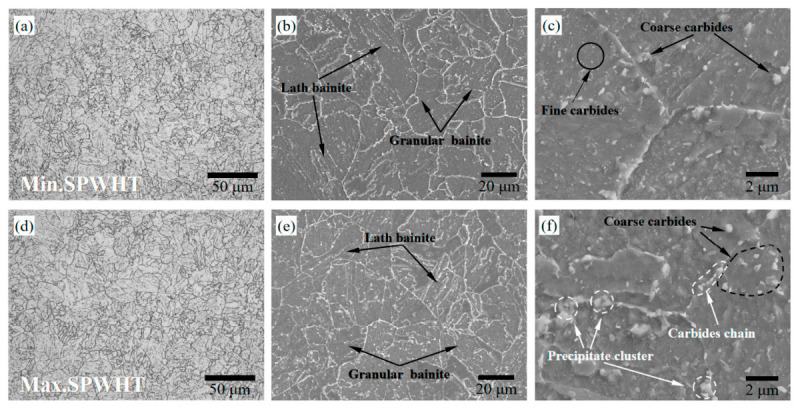
Microstructures of 2.25Cr-1Mo-0.25V steel after simulated post-weld heat treatment (SPWHT). (**a**) Optical microstructure after minimum SPWHT; (**b**) low-magnification SEM image after minimum SPWHT showing lath bainite and granular bainite; (**c**) high-magnification SEM image after minimum SPWHT showing fine and coarse carbides; (**d**) optical microstructure after maximum SPWHT; (**e**) low-magnification SEM image after maximum SPWHT showing lath bainite and granular bainite; (**f**) high-magnification SEM image after maximum SPWHT showing coarse carbides, carbide chains, and precipitate clusters [7].

However, bainite in these steels should not be regarded as a single uniform microstructural entity. In heavy-wall sections, the actual microstructure may comprise bainitic ferrite, granular bainite, lath bainite, martensite–austenite (M/A) constituents, retained austenite with different stability, and multiple carbide populations whose morphology and distribution continue to evolve during tempering. This complexity is particularly important in large forgings because the slower cooling rate in the core tends to promote granular bainite and relatively coarse M/A constituents, whereas the faster-cooled near-surface regions are more likely to develop finer lath-type products. As illustrated in Figure 6, metallographic observations of 2.25Cr-1Mo-0.25V steel after minimum and maximum simulated post-weld heat treatment (PWHT) show bainite-dominated microstructures in both conditions, but they also reveal differences in lath coarsening and carbide precipitation, especially along boundaries and in clustered regions [7]. These observations demonstrate that bainitic morphology and carbide evolution in this steel family are strongly dependent on section thickness, cooling history, and subsequent heat treatment. Representative simulations of central and surface regions in industrial heavy-wall forgings further show that normalized conditions are associated mainly with granular bainite, whereas oil-quenched conditions are closer to lath bainite, confirming the pronounced thickness dependence of bainitic morphology in Cr-Mo-V reactor steels [7,16,17,33,34,35,36,37].

The significance of this mixed bainitic microstructure lies in the fact that the bainitic matrix alone does not determine the final properties. The fraction, morphology, and stability of M/A constituents, together with the presence of retained austenite, strongly influence strength, ductility, impact toughness, and the ductile-to-brittle transition behavior. In particular, retained austenite and M/A islands are not merely passive secondary features: their subsequent decomposition during tempering can markedly alter carbide precipitation behavior and thus change the toughness response. For 2.25Cr-1Mo-0.25V heavy forgings, retained austenite in the as-quenched granular bainite has been shown to decompose during tempering at 700 °C into ferrite and coarse M23C6 carbide clusters, and this coarse clustered precipitation can significantly deteriorate impact toughness [16,17,29,35,36,37].

This point is highly relevant for hydrogen-service steels because microstructural heterogeneity affects not only mechanical-property scatter, but also hydrogen interaction with the matrix and precipitates. Previous work has shown that carbide evolution in bainitic 2.25Cr-1Mo-0.25V steels changes hydrogen trapping behavior and contributes to differences in hydrogen permeation and hydrogen-related degradation after thermal exposure. Therefore, in 12Cr2Mo1V heavy-wall forgings, the merit of a bainitic microstructure should be evaluated not simply by whether bainite is present, but by whether the bainite-related constituents—including substructure scale, M/A morphology, retained-austenite stability, and carbide state—are all maintained within a favorable window across the entire section [20,26,27,28].

A more quantitative assessment of bainitic structure should combine several characterization scales rather than rely only on optical or SEM morphology. EBSD is useful for prior-austenite grain reconstruction, packet/block or effective-grain-size evaluation, high-angle boundary statistics, local misorientation/KAM mapping, and approximate quantification of M/A constituent distribution when the step size is sufficiently small. TEM and high-resolution STEM/EDS are required to identify nanoscale carbides, dislocation substructure, fine retained austenite, and carbide/matrix interfaces that are normally below the reliable resolution of EBSD. Modern 3D reconstruction methods, including serial sectioning, FIB-SEM tomography, and X-ray or diffraction-based approaches, can further reveal the connectivity and spatial distribution of M/A islands, carbide clusters, segregation bands, and residual defects. Therefore, EBSD provides statistical crystallographic information, TEM provides phase/precipitate verification, and 3D methods provide spatial connectivity; none of these methods alone are sufficient for fully evaluating thick-section bainitic heterogeneity [15,26,29,34,35,36,37,38,39,40].

The literature also contains an apparent contradiction regarding retained austenite. In some bainitic steels, stable film-like retained austenite can improve toughness by crack blunting, strain accommodation, and transformation-induced plasticity; in heavy Cr-Mo-V reactor steels, however, blocky or unstable retained austenite and M/A constituents may decompose during tempering into ferrite plus coarse M23C6-rich carbide clusters, thereby reducing impact toughness. This contradiction can be reconciled by considering retained-austenite morphology, carbon enrichment, stability, local constraint, and tempering path. For 12Cr2Mo1V shell forgings, retained austenite should therefore be evaluated not only by volume fraction, but also by morphology, carbon stability, spatial location, and its subsequent carbide-forming tendency during tempering or PWHT [16,17,29,34,35,36,37,41,42,43].

The practical implication is that microstructure control, rather than nominal composition alone, ultimately determines whether the advantages of low-carbon bainite can be translated into stable section-wide performance. When bainitic morphology is refined and reasonably uniform, and when M/A constituents and carbides are properly regulated by heat treatment, the steel can achieve an advantageous strength–toughness combination together with better stability for high-temperature and hydrogen-bearing service. By contrast, if local hard constituents, coarse carbide clusters, or inherited through-thickness heterogeneity are retained, the intrinsic benefit of the bainitic design may be offset, leading to significant scatter in toughness and service reliability. This is why the low-carbon bainitic microstructure should be viewed as a controlled microstructural system rather than a simple phase label [7,16,17,26,29].

### 2.3. Material Advantages for Heavy-Wall Shell Forgings

For heavy-wall hydrogenation reactor shells, the principal advantage of 12Cr2Mo1V lies in its ability to provide a suitable combination of hardenability, tempering resistance, elevated-temperature strength, and hydrogen-service reliability. In the broader literature on closely related 2.25Cr-1Mo-0.25V reactor steels, V addition is consistently associated with improved high-temperature strength, improved resistance to temper embrittlement and hydrogen-related degradation, and adequate toughness for severe pressure-vessel service. These features are especially valuable for large-shell forgings, in which long-term service stability is required under the simultaneous action of high temperature, high pressure, and hydrogen-bearing media [20,21,23,24,25,26,44,45,46,47].

Another important advantage is that the alloy is compatible with bainite-dominated microstructures that are more realistic for thick sections than fully martensitic structures. This point is metallurgically significant because the large cross-sections of reactor shells make uniform rapid cooling inherently difficult. In related heavy-wall 2.25Cr-1Mo-0.25V steels, the surface and core have been shown to develop different initial bainitic morphologies under different cooling conditions, with lath bainite being favored in faster-cooled regions and granular bainite being more typical of slower-cooled regions. Such behavior indicates that the Cr-Mo-V alloy design offers a relatively tolerant microstructural basis for massive forgings, provided that heat treatment is properly controlled. At the same time, available studies also show that this tolerance is not unlimited, because tempering response remains sensitive to the initial bainitic state, the persistence or decomposition of M/A constituents, and the evolution of carbide populations [7,16,18,19,22,29,33].

However, the material-level advantages of 12Cr2Mo1V do not eliminate the core metallurgical difficulties associated with super-thick forgings. First, section-size effects remain unavoidable: temperature history, deformation degree, and cooling rate vary substantially from the surface to the core, which can lead to through-thickness gradients in bainitic morphology, carbide evolution, and final properties. Second, the effective heat-treatment window is relatively sensitive. Even modest changes in austenitization, cooling path, or tempering schedule may alter retained-austenite stability, M/A decomposition behavior, and the precipitation state of carbides, thereby affecting both toughness and long-term service stability. For this reason, the advantage of the alloy should not be understood as an automatic guarantee of performance, but rather as a favorable metallurgical potential that must be realized through controlled processing [23,27,29,33,48,49,50].

More importantly, internal quality and microstructural uniformity cannot be separated in heavy-wall shell forgings. Free forging is expected not only to shape the component but also to consolidate inherited casting defects and improve internal soundness. Studies on heavy ingots and large free-forged products show that residual voids, porosity-related defects, macrosegregation, and insufficient central deformation remain critical limiting factors when deformation conditions are inadequate. Therefore, for 12Cr2Mo1V shell forgings, the key challenge is not whether the alloy possesses attractive intrinsic properties, but whether those properties can be reproduced consistently across the full wall thickness under industrial manufacturing conditions [9,10,11,12,13,14,15,51,52,53,54,55,56,57,58,59,60,61,62,63].

In this sense, the major merit of 12Cr2Mo1V for hydrogenation reactor shells is best described as a combination of metallurgical suitability and process compatibility: the alloy offers a favorable basis for thick-section bainitic microstructures and high-temperature hydrogen service, but its full advantage can only be achieved when forging densification, heat-treatment control, and through-thickness microstructural homogenization are all properly coordinated [27,29,33,48,49,64].

## 3. Free Forging and Defect Control

### 3.1. Manufacturing Route of Shell Forgings

**Figure 7:**
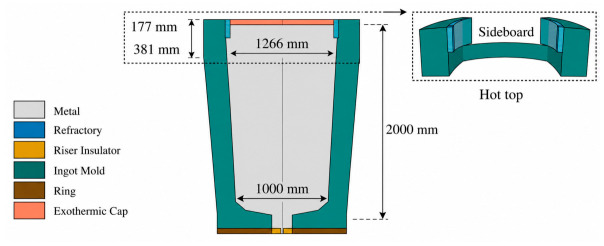
Redrawn casting assembly for a 12 MT steel ingot, showing the mold, hot top, refractory/insulation system, riser, and exothermic cap at the starting-stock stage before free forging. Adapted and modified from Ref. [14].

In this review, free forging is used in the same technical sense as open-die forging in the international forging literature. It denotes a sequence of operations such as cogging, upsetting, drawing, piercing or hollowing, mandrel-assisted expansion, and shell forming, rather than closed-die impression forging. The internal quality problem therefore begins at the ingot stage rather than in the final shell-forming steps. As shown in Figure 7, the casting assembly of a large steel ingot, including the mold and hot top, determines the thermal and feeding conditions that influence shrinkage, segregation, and other inherited inhomogeneities in the starting stock [14]. Although the exact forging sequence varies with ingot type, equipment capacity, and shell dimensions, its metallurgical objective is consistent: the route must transform a large cast starting stock into a sound thick-wall shell with adequate internal consolidation, weakened inherited heterogeneity, and a controllable heat-treatment response [65,66,67,68].

**Figure 8:**
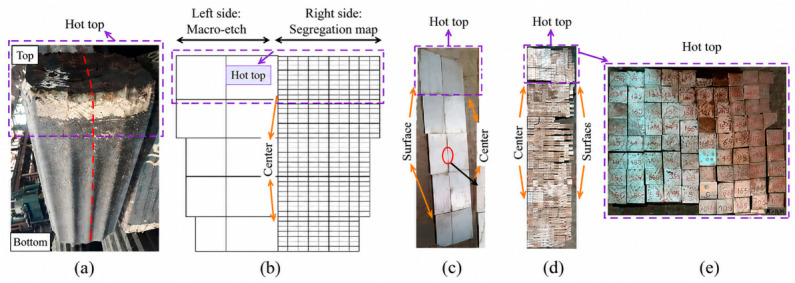
Sectioning, macro-etching, and chemical-mapping workflow for a 12 MT cast steel ingot. (**a**) As-cast ingot showing the hot top region, top-bottom direction, and central longitudinal sectioning position; (**b**) sampling layout, with the left side used for macro-etching and the right side divided into a grid for segregation mapping; (**c**) longitudinal macro-etched section showing the center-to-surface sampling direction; (**d**) sectioned samples for characterizing macrosegregation along height and radial directions; and (**e**) chemical-mapping blocks from the hot top region. Adapted and modified from Ref. [14].

The early breakdown stage is one of the most critical parts of the route because it determines whether the cast structure is sufficiently worked before the final hollow shell geometry is generated. In heavy free-forging practice, cogging and upsetting are not merely preparatory shaping steps; they are the principal operations used to introduce strain into the interior, promote hydrostatic compression, and reduce the detrimental influence of casting-derived defects. As shown in Figure 8, investigations of large cast steel ingots often use central longitudinal sectioning, macro-etch sampling, and chemical mapping to reveal segregation and other inherited inhomogeneities across the ingot [14]. These observations make clear that macrosegregation and shrinkage porosity are persistent quality-limiting features and that their effects may remain unless the early forging schedule provides sufficient deformation penetration into the core. Therefore, the metallurgical significance of early breakdown lies in disrupting the cast structure, improving internal soundness, and establishing a more favorable precursor for subsequent shell-forming and heat treatment [51,52,53,54,55,61,62,63].

For this reason, the shell-forging route cannot be designed as a purely geometric forming path. Process variables such as die shape, die-width ratio, reduction per pass, bite ratio, feed strategy, rotation schedule, reheating practice, and inter-pass temperature affect the distribution of effective strain and compressive stress in the workpiece interior. Studies on free forging show that void closure and core working are highly sensitive to these variables, and local effective strain is often used as an indicator of whether internal voids can be eliminated. A commonly cited simulation-based criterion suggests that a local effective strain of approximately 0.6 or higher is required for reliable void closure during hot free forging of large cast ingots [53,54,55,57,58,59,60,61,62,63,65,67].

From the viewpoint of route comparison, conventional free forging remains the most flexible method for very large shell forgings because it can accommodate large ingots, variable reductions, reheating cycles, and progressive geometry changes. Its limitation is that strain penetration and void closure are highly dependent on pass design and operator-controlled bite/rotation schedules. Multi-axis deformation routes improve this situation by changing the loading direction and strain path, thereby increasing the probability of core working and reducing directional heterogeneity, but they require stricter control of temperature, pass sequence, and equipment capacity. Radial forging can provide more symmetric multi-directional compression and better dimensional repeatability for shafts, tubes, and hollow preforms; however, its application to ultra-large reactor shells is constrained by workpiece size, mandrel design, tooling rigidity, and the need to preserve internal-quality improvement during subsequent shell expansion. Automated deformation control, based on press stroke, load, temperature, geometry measurement, and FEM/surrogate feedback, is therefore valuable because it can transform empirical pass scheduling into repeatable core-strain and defect-closure control [51,52,53,54,55,56,57,58,59,60,61,62,63,65,66,67,68].

In shell forgings, process design becomes more demanding because cylindrical hollow geometry must be generated without sacrificing the center-quality improvement achieved during breakdown. Once the operation proceeds from solid-stock consolidation to piercing, hollowing, mandrel expansion, or shell enlargement, the opportunity for further improving the most defect-prone central region becomes narrower. If the early cogging or breakdown schedule is insufficient, later shell-forming steps may achieve the required dimensions while leaving the interior underworked and through-thickness quality nonuniform. The manufacturing route of reactor shell forgings should therefore be understood as a coupled process of shaping and internal quality engineering rather than as a sequence of isolated forming operations [51,53,56,59,60,61,62,63].

In this sense, free forging is the pivotal stage of the shell-manufacturing route. It determines not only the macroscopic shell geometry but also whether subsequent heat treatment acts on a dense and reasonably homogeneous forged precursor or on a material state that still contains residual internal defects, segregation-related heterogeneity, and strong through-thickness differences inherited from casting and deformation history. Thus, in a review of 12Cr2Mo1V heavy-wall shell forgings, the manufacturing route should be discussed primarily as the foundation for defect mitigation and microstructural conditioning rather than simply as a forming sequence [51,52,53,54,55,56,57,58,59,60,61,62,63].

### 3.2. Internal Defects in Heavy-Section Forgings

**Figure 9:**
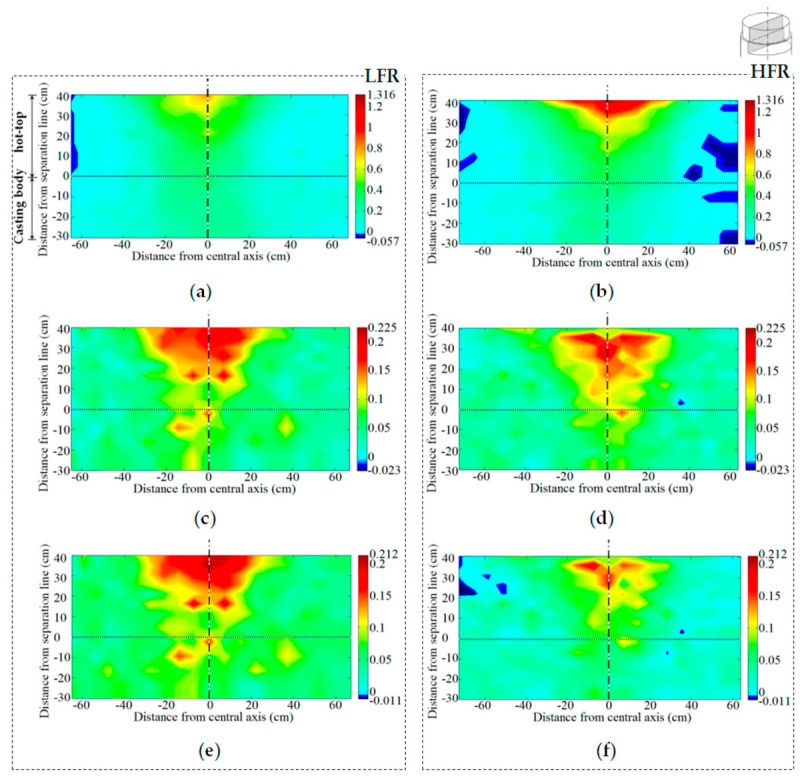
Chemical segregation-ratio patterns in low-filling-rate (LFR) and high-filling-rate (HFR) steel ingots. (**a**) C distribution under LFR; (**b**) C distribution under HFR; (**c**) Mn distribution under LFR; (**d**) Mn distribution under HFR; (**e**) Cr distribution under LFR; (**f**) Cr distribution under HFR [8].

Because hydrogenation reactor shell forgings are generally produced from heavy ingots or large cast stocks, their internal quality is strongly conditioned by casting-derived defects inherited at the starting stage. The most important defects include shrinkage cavities, centerline shrinkage porosity, microporosity, macrosegregation, and non-metallic inclusions; large cast ingots may also retain pronounced as-cast structural heterogeneity before sufficient breakdown deformation is imposed. Reviews on large steel ingots emphasize that macrosegregation and shrinkage porosity are among the principal factors limiting the homogenization of large cast and forged products, while investigations of industrial-scale Cr-Mo ingots show that such defects often coexist within the same starting stock rather than appearing as isolated imperfections [9,10,11,12,13,14,15]. Macrosegregation is especially important because it represents chemical heterogeneity at the ingot scale and changes the local composition inherited by subsequent forging and heat treatment. As illustrated in Figure 9, segregation-ratio maps for C, Mn, and Cr under low and high filling rates provide an example of how ingot-scale positive and negative macrosegregation can persist as inherited heterogeneity after casting and subsequent processing [8,69]. In a 12 MT Cr-Mo steel ingot, experimental mapping revealed positive segregation near the top region, conical negative segregation near the bottom, and a solute-enriched zone between the center and the ingot wall. Such heterogeneity is difficult to eliminate completely, even by prolonged homogenization, and can degrade mechanical performance, structural uniformity, and machinability of the final component [9,10,11,12,13,14].

For thick-wall shell forgings, these inherited defects are further amplified by the section-size effect. The surface and core regions do not experience the same deformation and thermal history during forging and heat treatment, and related studies on 2.25Cr-1Mo-0.25V heavy-wall reactor steels have shown that the center and surface regions can develop markedly different initial microstructures because of different cooling rates during quenching. At the same time, free-forging studies on large cast ingots indicate that internal voids must be eliminated during the early cogging or upsetting stages; otherwise, they may persist into later stages and act as defect sources during subsequent processing. In other words, the center of the workpiece is both the region most vulnerable to inherited defects and the region most difficult to improve effectively [9,13,14,15].

The practical consequence is that internal defects in heavy-section shell forgings should be evaluated in terms of both defect severity and property uniformity. For hydrogenation reactor shells, through-thickness consistency is not a secondary quality index but a core performance requirement. Once inherited defects, chemical segregation, and nonuniform deformation are superimposed, the result is not only a reduction in average toughness or strength, but also a widening of performance scatter across the wall thickness. This is consistent with observations in related heavy-wall Cr-Mo-V steels, where microstructural differences between surface-simulated and center-simulated regions led to different strength and ductile-to-brittle transition responses during tempering [9,15,33].

### 3.3. Defect Closure During Free Forging

One of the most important metallurgical functions of free forging is the closure and consolidation of internal defects inherited from large cast starting stocks. Defect closure, however, is not governed simply by the nominal forging ratio. Classical simulations and recent experimental studies on heavy forgings show that successful healing depends on the combined action of compressive stress state, effective strain, strain path, temperature, and contact conditions at the defect surfaces. High compressive triaxiality promotes void collapse, whereas sufficient local deformation is required to ensure intimate contact, interfacial bonding, and permanent elimination of the defect. Consequently, nominally similar reductions may lead to different closure efficiencies when die geometry, bite ratio, pass schedule, or deformation sequence changes. Several studies therefore use local deformation or void-closure criteria, including the commonly cited effective-strain threshold of approximately 0.6, to evaluate hot free forging of large ingots [51,52,53,54,55,56,57,58,59,60,61,62,63].

For reactor shell forgings, this understanding has two direct implications. First, early breakdown and cogging are decisive because they provide the best opportunity to impose compressive strain deep into the interior before the workpiece geometry becomes increasingly specialized. If the primary breakdown stage is insufficient, later shell-forming operations may meet dimensional requirements but leave the central region underworked. Second, defect closure is defect-type dependent. Small dispersed porosity can often be consolidated effectively, whereas large shrinkage cavities, segregation-associated channels, and inclusion-related damage are much more difficult to eliminate completely. Free forging should therefore be regarded as a powerful densification process, but not as a universal method for removing all casting-derived heterogeneities [51,52,53,54,55,61,62,63]. This interpretation is consistent with work by Kwon et al., who reported ultrasonic confirmation of cavity closure at a forging ratio of approximately 2.9S in large steel ingots.

Another point that deserves emphasis in a review of heavy-wall shell forgings is that defect closure must be evaluated together with microstructural evolution rather than as an isolated geometric event. Experimental work on forging healing has shown that the restoration of tensile properties can occur once porosity defects are effectively healed, but the recovery of impact and fatigue properties requires a more advanced state in which the joint region and the surrounding matrix also become microstructurally homogenized. In other words, the elimination of a visible pore does not necessarily mean that the local metallurgical weakness has disappeared. Repeated upsetting and drawing are often needed not only to eliminate internal defects, but also to remove flat grain bands, promote recrystallization or grain-boundary migration, and reduce the microstructural discontinuity between the healed zone and the surrounding matrix [15,61,62].

This point is especially important for hydrogenation reactor shells, where the engineering target is not merely to reduce the number of internal voids but to produce a section that is both dense and sufficiently homogeneous for subsequent heat treatment. Excessively surface-biased deformation may improve external shape while leaving the core incompletely healed. By contrast, a route that achieves favorable strain penetration and compressive stress distribution can simultaneously promote densification, weaken the structural consequences of casting heterogeneity, and establish a better basis for grain refinement, bainitic transformation control, and through-thickness property uniformity. The real measure of forging success in reactor shell manufacture is therefore not the forging ratio recorded on the process sheet alone, but the extent to which free forging improves center quality and prepares a uniform metallurgical precursor for final heat treatment [51,52,53,54,55,56,57,58,59,60,61,62,63]. The main process variables and their metallurgical consequences are summarized in Table 2.

## 4. Microstructural Evolution During Forging and Heat Treatment

### 4.1. Microstructural Evolution During Hot Deformation

**Figure 10:**
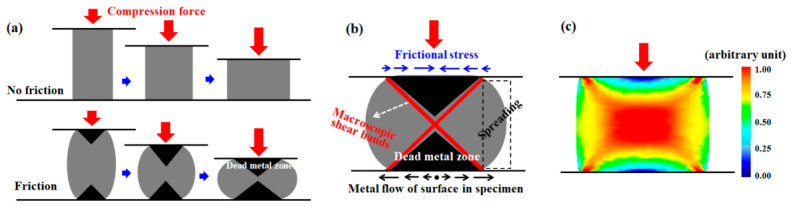
Contact-pressure and friction effects during compression-type bulk forming. (**a**) Material flow under no-friction and frictional compression; (**b**) friction-induced material flow, macroscopic shear bands, and dead-metal-zone formation; (**c**) representative simulated strain distribution in a compressed specimen [48].

During free forging, the initial cast structure is progressively broken down and replaced by a thermomechanically conditioned austenitic structure that serves as the precursor for subsequent phase transformation and tempering. The essential metallurgical processes include dissolution or redistribution of inherited precipitates, dislocation multiplication and rearrangement, dynamic recovery, dynamic recrystallization (DRX), and grain-boundary migration. As illustrated in Figure 10, compression-type bulk-forming studies show that die friction and contact pressure can generate nonuniform material flow, dead-metal zones, and localized strain fields, which are important when interpreting hot-compression data and forging simulations [48]. Related hot-deformation studies on Cr-Mo steels commonly employ controlled routes involving heating, short holding, hot compression, and rapid quenching, thereby providing a thermomechanical basis for analyzing constitutive behavior and recrystallization response [12,37,49]. Experimental results obtained over 900–1200 °C and 0.01–5 s^−1^ show that both flow behavior and the final deformed microstructure are highly sensitive to deformation temperature and strain rate. These findings confirm that hot deformation in 12Cr2Mo1V steel is not merely a geometric shaping stage but a microstructural renewal process that strongly influences the austenitic precursor state and the subsequent evolution of final microstructure and properties [49,70,71,72].

**Figure 11:**
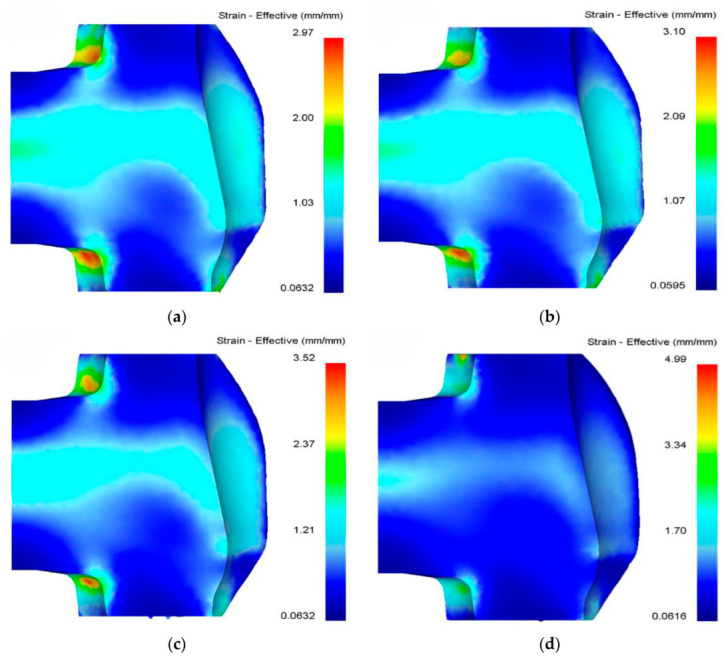
Effective-strain distributions of 25CrMo4 steel during deformation at different temperatures. (**a**) 950 °C; (**b**) 1000 °C; (**c**) 1050 °C; (**d**) 1100 °C [49].

For reactor shell forgings, the key requirement of hot deformation is not only sufficient shape change but also adequate internal structural renewal. Recent studies on related Cr-Mo steels show that higher deformation temperature and lower strain rate generally promote DRX and reduce geometrically necessary dislocation (GND) density, whereas unfavorable parameter combinations cause incomplete recrystallization, subgrain accumulation, and persistent structural heterogeneity. As illustrated in Figure 11, finite-element deformation simulations show that effective-strain distributions remain nonuniform even under controlled compression conditions, directly affecting local recrystallization and final microstructural uniformity [49]. The same study identified an optimal hot-working window of approximately 1170–1200 °C and 0.01–0.1 s^−1^. In contrast, 900 °C/5 s^−1^ produced elongated prior-austenite grains and adiabatic shear bands, and 1100 °C/5 s^−1^ still produced mixed grain sizes because recrystallization remained incomplete. These findings are relevant to heavy shell forgings because process design must ensure that the core is deformed under conditions favorable for austenite renewal and homogenization.

Another important feature of industrial free forging is that it is an intermittent thermomechanical process rather than a single-pass compression event. Between passes, the workpiece undergoes surface cooling, internal heat conduction, reheating, and redistribution of local strain. As a result, the austenitic structure prior to quenching or normalizing is usually heterogeneous across the section, especially in heavy-wall products. This interpretation is consistent with observations in related 2.25Cr-1Mo-0.25V heavy-wall steels, in which surface-simulated and center-simulated regions developed different initial bainitic morphologies owing to different thermal histories, and these differences subsequently affected tempering response and property evolution. Accordingly, the metallurgical effect of hot deformation in shell forgings should be evaluated in terms of the austenitic condition established for later transformation, rather than only in terms of instantaneous deformation resistance [15,49,65,66,70,72].

The practical implication is that forging and heat treatment should be treated as a coupled sequence rather than two independent operations. If hot deformation fails to produce a sufficiently renewed and reasonably uniform prior-austenite structure, subsequent quenching, normalizing, or tempering will act on a heterogeneous starting state and may amplify through-thickness differences in bainitic morphology, carbide evolution, and final mechanical performance. For 12Cr2Mo1V heavy-wall shell forgings, the hot-working window must therefore be selected not only for forgeability and load control, but also for its ability to support later microstructural uniformity and stable section-wide properties [49,65,66,67,68,70,71,72].

### 4.2. Bainite, Grain, and Carbide Evolution

**Figure 12:**
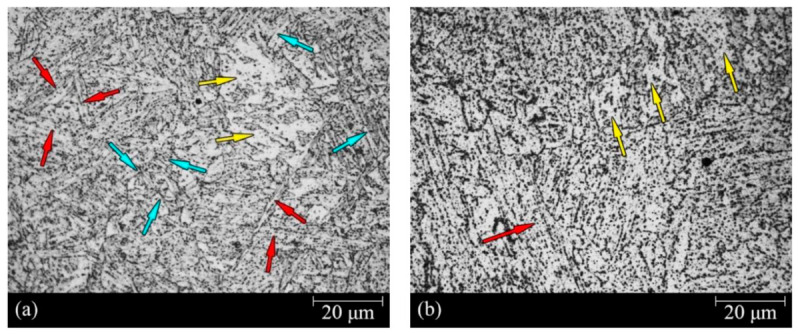
Optical microstructures of 2.25Cr-1Mo-0.25V steel. (**a**) Raw steel showing acicular ferrite, quasi-polygonal ferrite, and lath ferrite features; (**b**) annealed steel showing a carbide-containing transformed matrix after annealing [26].

After hot deformation, the final microstructure of 12Cr2Mo1V heavy-wall shell forgings is established through the combined effects of cooling path and tempering response. In thick sections, this evolution is inherently nonuniform because the cooling rate decreases progressively from the surface toward the core. Consequently, the near-surface region is more likely to develop finer lath-type bainitic products, whereas the more slowly cooled interior tends to form coarser granular bainite together with a higher fraction of martensite–austenite (M/A) constituents. Studies on related 2.25Cr-1Mo-0.25V heavy-wall reactor steels have directly shown that surface-simulated and center-simulated regions can begin with different bainitic morphologies, and that these initial differences remain important during subsequent tempering. As illustrated in Figure 12, the optical microstructures of raw and annealed 2.25Cr-1Mo-0.25V steel exhibit features such as acicular ferrite, quasi-polygonal ferrite, and lath ferrite, providing a useful visual example of the microstructural heterogeneity that may develop in this class of steels under different thermal histories [26]. This surface-to-core transformation gradient is one of the main reasons why thick-shell forgings may display substantial through-thickness variation even when they are produced from the same heat and nominal forging route [14,38].

Grain size and microstructural scale remain fundamental control variables, but they should not be considered in isolation. A finer transformed structure generally promotes toughness and suppresses local brittle response, whereas coarse prior-austenite grains, wide laths, and coarse bainitic packets tend to reduce crack-initiation resistance. In vanadium-modified Cr-Mo reactor steels, however, grain refinement alone does not fully determine performance, because carbide evolution during tempering is equally important. Available studies show that several carbide types may appear, transform, or coarsen in this steel family, including M3C, M7C3, M23C6, M2C, and MX/MC-type precipitates, and their relative fractions depend strongly on the initial microstructure and thermal history. Their size, chemistry, and spatial distribution influence precipitation strengthening, tempering stability, hydrogen trapping behavior, and fracture characteristics [16,17,18,19,22,30,31,32,38,39].

The behavior of M/A constituents and retained austenite is particularly important in bainite-dominated heavy-wall steels. In as-quenched granular bainite, retained austenite and blocky M/A islands may survive in the microstructure, but their later decomposition during tempering can have opposite effects depending on the temperature window and the resulting precipitation mode. A representative study on 2.25Cr-1Mo-0.25V heavy forgings showed that retained austenite in granular bainite decomposed during tempering at 700 °C into ferrite and coarse M23C6 carbide clusters, and that this clustered carbide morphology significantly deteriorated impact toughness. By contrast, when the decomposition pathway was modified through pre-tempering, finer transitional structures were produced and the subsequent carbide distribution became less harmful. This means that the effect of M/A constituents is not simply controlled by their presence or absence, but by how they evolve into tempered products and precipitate assemblies [29,34,35,36,37,41,42,43].

**Figure 13:**
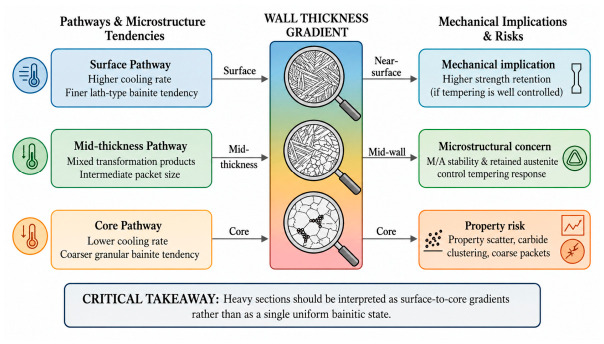
Through-thickness microstructural evolution in heavy-wall shell forgings, showing surface, mid-thickness, and core pathways controlled by cooling-rate differences, bainite morphology, M/A constituents, and carbide-related risks.

For this reason, the final performance of heavy-wall shell forgings is better understood in terms of a coupled microstructural triad consisting of bainite morphology, grain/substructure scale, and carbide state. A bainitic microstructure is beneficial only when these three factors evolve in a coordinated manner across the section thickness. As summarized in Figure 13, heavy-wall shell forgings should be interpreted as surface-to-core gradients rather than as a single uniform bainitic state: the near-surface region tends to follow a higher-cooling-rate pathway and develop finer lath-type bainite, the mid-thickness region commonly exhibits mixed transformation products, and the core more often shows a lower-cooling-rate pathway with coarser granular bainite. These through-thickness differences lead in turn to different mechanical implications and microstructural risks, including strength retention near the surface, tempering sensitivity in the mid-wall region, and property scatter associated with coarse packets, carbide clustering, and related heterogeneity in the core. Therefore, the advantage of a bainitic design can easily be offset if bainitic packets coarsen excessively, if retained-austenite-derived islands decompose into locally harmful carbide clusters, or if grain-boundary carbides become coarse and nonuniform. In 12Cr2Mo1V shell forgings, the key metallurgical task is thus not merely to “obtain bainite”, but to regulate the entire bainite–grain–carbide system so that it remains stable, well-tempered, and reasonably uniform after heat treatment [38,39].

### 4.3. Heat-Treatment Regulation of Final Microstructure

Heat treatment is the final decisive operation that converts the forged structure into a qualified service microstructure, but its role is better understood as selective regulation rather than complete reconstruction. For 12Cr2Mo1V heavy-wall shell forgings, heat treatment is expected to reduce microstructural heterogeneity as far as section size permits, regulate the relative fractions of bainitic and martensitic transformation products, decompose unstable constituents such as retained-austenite-derived M/A islands, control carbide precipitation and coarsening, and relieve stresses introduced during forging. In thick sections, however, the same nominal heat-treatment schedule does not necessarily produce the same metallurgical response throughout the wall thickness, because the starting microstructure entering heat treatment is already heterogeneous [7,16,17,19,22,29,64].

This section-size sensitivity is well illustrated by the literature on vanadium-modified reactor steels. In large-thickness 2.25Cr-1Mo-0.25V steel subjected to simulated PWHT, longer thermal exposure reduced strength, hardness, and toughness, while also promoting the degradation of lath bainite, the relative increase in granular bainite, and the precipitation, spheroidization, and coarsening of carbides, including chain-like carbide clusters at longer holding times. These results show that heat treatment is not simply a stress-relief step; it actively changes the balance among bainite morphology, precipitate state, and toughness. Accordingly, any discussion of heat-treatment optimization for shell forgings must address both transformed-matrix evolution and carbide stability [16,17,19,22,23,27,29,48,49,50,73].

A second key point is that different initial microstructures do not converge to an identical final condition under the same tempering schedule. In a study designed to simulate the center and surface regions of heavy-wall 2.25Cr-1Mo-0.25V forgings, normalized material with granular bainite and oil-quenched material with lath bainite exhibited different strength levels and different ductile-to-brittle transition temperature (DBTT) responses during tempering at 700 °C. The normalized condition showed a more pronounced decrease in strength and DBTT with increasing tempering time, which was attributed to decomposition of M/A constituents and growth of VC-type precipitates, whereas the oil-quenched condition evolved differently because of its finer lath-based starting structure. This finding is highly relevant for heavy shell forgings because tempering cannot be assumed to erase the surface-to-core transformation gradient created during quenching or normalizing [16,17,29,33,64].

Unintended exposure to intercritical temperatures is another important concern. A recent open-access study on 2.25Cr-1Mo pressure-vessel steel showed that intercritical quenching and tempering produced a dual-phase microstructure containing ferrite and tempered martensite, together with Cr-Mo-enriched carbides, and this condition led to marked losses in yield strength, ultimate tensile strength, and toughness compared with conventional quench-and-temper treatment. Although this work was performed on 2.25Cr-1Mo rather than the vanadium-modified grade, its implication is directly relevant here: localized intercritical thermal excursions during fabrication, repair, or complex thermal cycling can generate unfavorable phase mixtures that degrade the final property balance instead of improving it [27,64].

**Figure 14:**
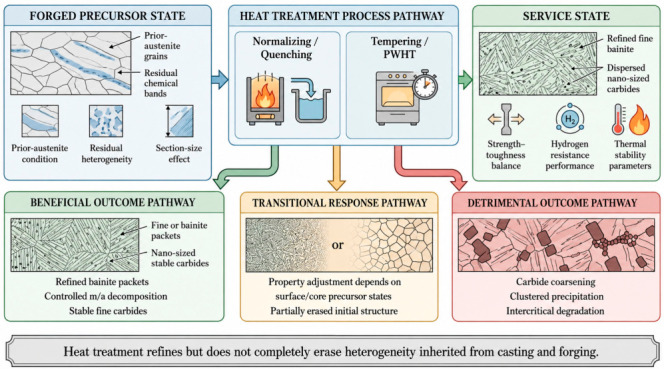
Heat-treatment regulation of the bainite–grain–carbide system and its linkage with mechanical response, transformation behavior, precipitation state, and service performance.

For heavy-wall shell forgings, heat treatment is most effective when it is designed specifically for the thickness-dependent precursor state created by forging. Its role is not simply to bring the material into compliance with nominal property requirements, but to regulate through-thickness microstructural uniformity, control the decomposition of hard constituents, and stabilize carbide evolution during long-term service. Figure 14 synthesizes this role by showing that the forged precursor state may evolve through normalizing/quenching and tempering/PWHT into different outcome pathways, ranging from beneficial refinement of bainite and fine carbide stabilization to transitional or even detrimental responses involving residual heterogeneity, carbide coarsening, and clustered precipitation. This perspective highlights an important limitation: although heat treatment can refine and optimize the forged structure, it cannot completely remove heterogeneity inherited from casting and forging. Accordingly, the literature indicates that the most reliable route for heavy shell forgings is an integrated forging–heat-treatment strategy, in which forging provides a sound and relatively uniform precursor condition and heat treatment converts it into a stable service microstructure and property state [27,29,30,31,32,33,64,70].

## 5. Mechanical Properties and Service Performance

### 5.1. Strength and Toughness

For hydrogenation reactor shell forgings, room-temperature strength and impact toughness remain the basic qualification indices, but the engineering requirement is more stringent than meeting a single set of nominal values. Because these components are thick-wall forgings, their properties must remain sufficiently stable across the wall thickness and from one local region to another. In vanadium-modified Cr-Mo reactor steels, strength is mainly governed by the transformed substructure, dislocation density, and precipitation state, whereas toughness is more sensitive to prior-austenite grain size, bainitic morphology, M/A constituents, coarse carbides, and residual internal defects. Strength and toughness should therefore be discussed together, but they should not be treated as equally sensitive indicators of shell-forging quality [16,17,20,21,26,44,45,51,67,68].

This point becomes particularly important in heavy-wall sections because microstructural inhomogeneity readily translates into property scatter. In a study on large-thickness 2.25Cr-1Mo-0.25V steel for hydrogenation reactors, increasing simulated PWHT duration led to a progressive decrease in hardness, −30 °C impact energy, yield strength, and tensile strength, demonstrating that the final strength–toughness balance is strongly affected by thermal history. In another study designed to simulate the center and surface regions of industrial heavy-wall forgings, normalized material with granular bainite and oil-quenched material with lath bainite showed different strength levels and different ductile-to-brittle transition responses during tempering, confirming that thickness-related microstructural differences do not simply disappear under the same tempering schedule [7,16,17,18,19,22,29,33].

To strengthen the quantitative comparison requested for heavy-wall forgings, the key evidence should be organized by sampling location, heat-treatment state, and directly measurable property indicators rather than by average strength alone. Table 3 summarizes the most relevant indicators for comparing surface and core regions, PWHT sensitivity, retained-austenite decomposition, and defect-closure effectiveness. The table is intended as a compact framework for interpreting available data and for standardizing future reports on 12Cr2Mo1V and related Cr-Mo-V shell forgings.

For this reason, a high average tensile strength cannot by itself be taken as evidence of a sound shell forging. A component may still satisfy nominal strength requirements while containing a central region with reduced impact toughness, elevated transition temperature, or greater local sensitivity to brittle fracture initiation. In practical terms, toughness is often the more revealing indicator because it responds more sharply to coarse bainitic products, segregated zones, coarse carbide clusters, and incompletely healed internal defects. Accordingly, the assessment of shell-forging quality should emphasize not only whether strength and toughness meet specification limits, but also whether they remain reasonably uniform across the section thickness [65,66,67,72].

The underlying structure–property relationship is therefore straightforward but important: better densification, finer and more uniform transformation products, and a more stable carbide distribution generally promote a better strength–toughness match, whereas internal defects, coarse grains, segregation, and unfavorable precipitate evolution act synergistically to reduce toughness and increase performance scatter. For heavy-wall hydrogenation reactor shells, the decisive question is not whether one location exhibits acceptable tensile properties, but whether the forged and heat-treated shell as a whole can maintain a reliable and consistent strength–toughness balance [7,16,17,33,40,45].

### 5.2. High-Temperature and Hydrogen-Service Performance

**Figure 15:**
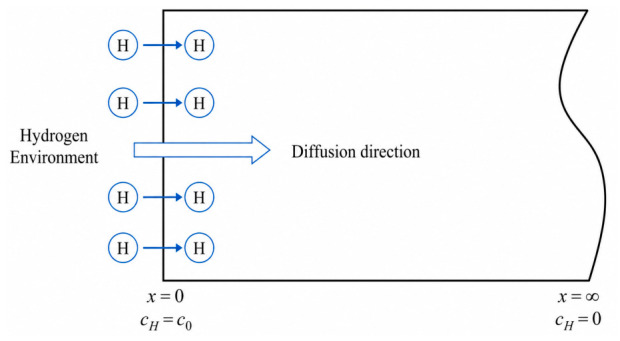
Redrawn and modified hydrogen-diffusion model for analyzing through-thickness hydrogen concentration evolution in 2.25Cr-1Mo-0.25V steel. Adapted and modified from Ref. [21].

Hydrogenation reactor shell forgings must satisfy a demanding combination of elevated-temperature strength, microstructural stability, and resistance to hydrogen-related degradation. Although vanadium-modified Cr-Mo steels were developed to meet these requirements, their service reliability depends not simply on nominal composition but on the final microstructural state produced by forging and heat treatment. Figure 15 summarizes this issue from the hydrogen-transport perspective by showing the one-dimensional diffusion model commonly used to describe hydrogen ingress and through-thickness concentration evolution in 2.25Cr-1Mo-0.25V steel [21]. This perspective highlights that hydrogen damage is controlled not only by total hydrogen uptake, but also by hydrogen redistribution and trapping within the section. Susceptibility to hydrogen embrittlement is strongly affected by dislocation structure, grain-boundary condition, carbide distribution, and trap character. Reported studies further show that cementite located at grain boundaries and lath ferrite interfaces behaves mainly as an irreversible hydrogen trap, indicating that hydrogen resistance is fundamentally a microstructure-dependent property. Therefore, the hydrogen-service performance of heavy-wall shell forgings should be understood as the outcome of coupled hydrogen diffusion, trap distribution, and microstructural stability rather than alloy chemistry alone [20,21,26,66].

**Figure 16:**
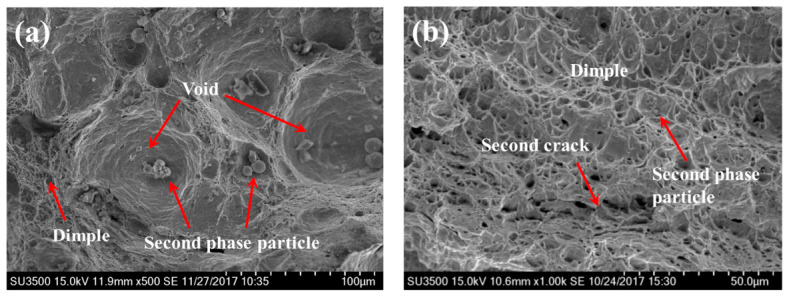
Representative fracture morphologies of 2.25Cr-1Mo-0.25V base-metal and weld-zone specimens under hydrogen-free testing conditions. (**a**) Base-metal fracture surface showing dimples, voids, and second-phase particles; (**b**) weld-zone fracture surface showing dimples, secondary cracks, and second-phase particles [45].

At elevated temperature and hydrogen pressure, an important degradation mode of reactor steels is high-temperature hydrogen attack (HTHA), in which hydrogen reacts with carbide-associated carbon and produces methane-filled internal damage. Reported studies on 2.25Cr-1Mo steels indicate that resistance to HTHA depends strongly on carbide stability and on the preceding bainitic or martensitic microstructure, with equilibrium M23C6 offering better resistance than cementite. Figure 16 provides a representative fracture example showing dimples, voids, secondary cracks, and second-phase particles in base-metal and weld-zone specimens [45]. Such features connect the microstructural state with actual fracture morphology after testing. For 12Cr2Mo1V heavy-wall shell forgings, this evidence emphasizes that service reliability is governed not only by the existence of carbides, but also by their type, distribution, and stability after forging, heat treatment, and long-term hydrogen exposure. Hydrogen-service behavior should therefore be evaluated as a microstructure-dependent damage process rather than as a purely compositional property [20,21,26,27,28,44,45].

At the same time, adequate load-bearing capability must be maintained at service temperature. The strengthening contribution of Cr, Mo, and V, together with the tempering stability provided by a properly regulated bainite–carbide system, is therefore essential. Yet the full advantage of this alloy family can only be realized when the shell forging is internally sound and reasonably uniform through the thickness. A chemically correct steel that still contains residual porosity, strong segregation, unstable tempered constituents, or locally unfavorable carbide morphologies remains vulnerable because those heterogeneities alter both hydrogen interaction and mechanical stability in service. Thus, the high-temperature and hydrogen-service performance of heavy shell forgings is best understood as the outcome of three coupled factors: alloy design, final microstructure, and internal quality [24,25,27,44,47].

### 5.3. Structure–Property Relationships

The preceding discussion can be condensed into a hierarchical structure–property framework for 12Cr2Mo1V heavy-wall shell forgings. At the first level, internal soundness provides the basis for reliable performance. Residual porosity, shrinkage-related defects, and severe macrosegregation reduce load-bearing continuity and increase the probability of local weak zones. Even when nominal composition and average tensile properties satisfy specification requirements, such inherited heterogeneities can still produce substantial scatter in impact toughness, transition behavior, and service reliability across the wall thickness. Internal quality should therefore be regarded as the first-level prerequisite for shell performance rather than a secondary manufacturing index [9,10,11,12,13,14,15,33,51,52,53,54,55,56,57,58,59,60,61,62,63].

The second level is transformation uniformity. Once gross defects are suppressed, shell performance depends strongly on whether the section develops a reasonably consistent bainitic microstructure after forging and heat treatment. A controlled bainite-dominated structure generally provides the most favorable balance between strength and toughness in thick-section Cr-Mo-V steels, but this benefit is conditional. If the surface and core retain markedly different transformation products, packet sizes, or M/A constituent distributions, their subsequent tempering responses also diverge, and the shell may exhibit acceptable average properties together with undesirable through-thickness performance gradients. The key issue is not simply whether bainite is present, but whether bainitic morphology and substructure scale are stable and reasonably uniform throughout the section [7,16,17,29,33].

The third level is precipitate regulation. In vanadium-modified Cr-Mo reactor steels, carbide state is a major determinant of final performance. Properly distributed fine carbides can support strength retention, tempering stability, and beneficial hydrogen trapping, whereas coarse grain-boundary carbides, clustered carbides derived from retained-austenite decomposition, or unstable precipitate populations can impair toughness and increase hydrogen-related susceptibility. In this sense, the bainite–grain–carbide system should be considered as a coupled microstructural unit: favorable performance is achieved only when transformation morphology, grain/substructure scale, and carbide evolution are simultaneously controlled [16,17,18,19,22,26,27,28,38,39].

Accordingly, the structure–property relationship of heavy-wall shell forgings is inherently hierarchical and process-dependent. Internal soundness forms the foundation, transformation uniformity defines section-wide consistency, and precipitate control determines whether strength, toughness, and hydrogen-service stability can be retained after tempering and during service. Only when all three levels are addressed together can the intrinsic metallurgical potential of 12Cr2Mo1V be translated into reliable shell performance. This is why forging and heat treatment do not merely influence performance in a general sense; they determine it by jointly controlling defect state, transformed microstructure, and precipitation condition [27,29,33].

## 6. Discussion, Challenges, and Perspectives

### 6.1. Internal Quality and Microstructural Uniformity

The most persistent bottleneck in 12Cr2Mo1V heavy-wall shell forgings is no longer the basic ability to manufacture large components, but the ability to do so reproducibly while maintaining internal soundness and microstructural uniformity across very large sections. Industrial control must suppress residual porosity, shrinkage-related defects, and severe segregation while minimizing through-thickness gradients in transformed microstructure and final properties. For the largest shell sections, the most difficult tasks remain centerline consolidation, segregation weakening, and stabilization of a sufficiently uniform grain- and packet-scale microstructure throughout the wall thickness. Large-ingot studies continue to show that macrosegregation and shrinkage porosity are key factors restricting the homogenization of large cast and forged products, especially when section size becomes extreme [9,10,11,12,13,14,15,33].

**Figure 17:**
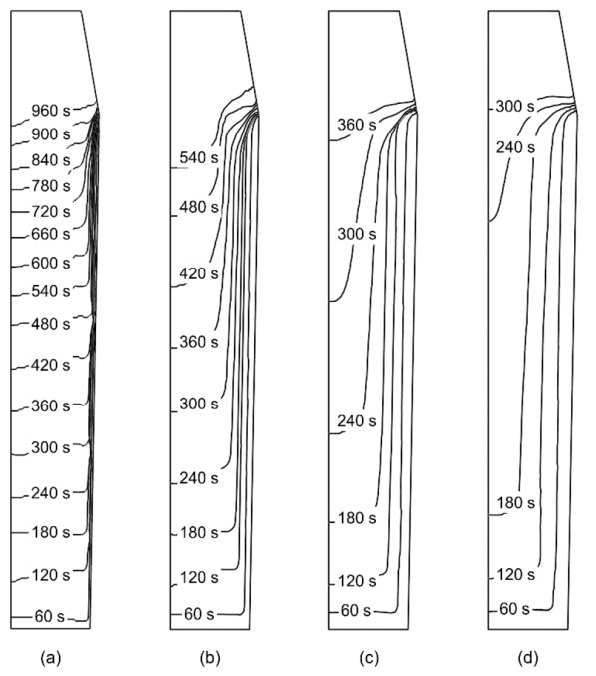
Packing-line evolution during steel-ingot solidification at different heat-transfer coefficients. (**a**) 100 W m^−2^ K^−1^; (**b**) 200 W m^−2^ K^−1^; (**c**) 300 W m^−2^ K^−1^; (**d**) 400 W m^−2^ K^−1^ [74].

This challenge is fundamentally multiscale. Macrosegregation originates during ingot filling and solidification; defect healing depends on local stress state, effective strain, and strain penetration during free forging; and the final transformation gradient is established during cooling and tempering because the surface and core do not experience the same thermal history. As illustrated in Figure 17, packing-line evolution under different cooling intensities in macrosegregation modeling shows how solidification-front development and central feeding behavior vary with heat-transfer conditions in thick ingots [74]. Because these stages are causally linked, isolated optimization of a single step usually yields limited improvement. A more aggressive pass schedule may enhance void closure, but it cannot eliminate chemistry-driven transformation differences inherited from segregation. Likewise, a refined heat-treatment schedule may reduce structural scatter, but it cannot fully compensate for an underworked core or severe casting-derived heterogeneity. Future quality control should therefore be based on an integrated casting–free-forging–heat-treatment perspective rather than isolated optimization of individual stages [9,10,11,12,13,14,15,51,52,53,54,55,56,57,58,59,60,61,62,63].

For this reason, future progress in heavy-wall shell forgings should treat internal quality and microstructural uniformity as a single integrated objective rather than as separate quality targets. In practical terms, this implies linking ingot-quality design, forging-route optimization, and thickness-oriented heat treatment into one coordinated process strategy. The key industrial challenge is not simply whether acceptable shells can be produced once, but whether the combined casting–forging–heat-treatment route can be controlled tightly enough to deliver low defect sensitivity, reduced through-thickness heterogeneity, and stable service performance from batch to batch [9,10,11,12,13,14,15,33,48,49].

### 6.2. Process Optimization of Forging and Heat Treatment

A clear direction for future process development is the transition from single-step optimization toward integrated forging–heat-treatment optimization. For 12Cr2Mo1V heavy-wall shell forgings, free-forging routes should not be judged only by dimensional feasibility or nominal forging ratio; they should also be assessed by their ability to promote core strain penetration, center-region compressive stress, defect closure, and a sufficiently uniform austenitic state before cooling or reheating. Recent work on 12Cr2Mo1V hot deformation shows that the hot-working window strongly influences dynamic recrystallization (DRX), dislocation density, and final structural uniformity, which means that the forging schedule directly affects the starting condition for subsequent heat treatment rather than merely the external shape of the shell [48,49,51,52,53,54,55,56,57,58,59,60,61,62,63,65,66,67,68].

The same integration logic applies to heat-treatment design. In thick-wall reactor steels, heat treatment should not be designed on the assumption of a homogeneous forged starting state, because the surface and core regions may enter tempering with different bainitic morphologies, M/A fractions, and precipitate distributions. Studies on 2.25Cr-1Mo-0.25V steel have shown that different initial microstructures respond differently to the same tempering schedule; therefore, a single nominal heat-treatment route does not automatically produce identical final properties throughout the section. In practical terms, optimal process design must couple the forging path with the intended heat-treatment response rather than using heat treatment as a last-stage corrective step [59,60,61].

Several process-development strategies are especially promising. The first is more effective deformation-path design during breakdown and shell forming so that the center receives greater strain penetration and more favorable hydrostatic compression. The second is the use of microstructure-based constitutive and transformation models to connect process parameters with final property gradients instead of optimizing only for shape or load. The third is tighter control of reheating, inter-pass temperature, and cooling history so that the forged austenitic structure enters quenching, normalizing, or tempering in a more uniform condition. More broadly, recent forging-optimization reviews emphasize that future progress depends on multi-objective, computer-aided engineering (CAE)-assisted design rather than isolated parameter tuning. The next generation of process optimization for 12Cr2Mo1V shell forgings should therefore focus on coordinated densification, homogenization, and transformation control rather than maximizing any single indicator in isolation [37,41,42,43,58,65,69,70,74,75].

### 6.3. Quantitative Comparison of Process and Prediction Approaches

A useful quantitative comparison of technological approaches should be based on common indicators rather than on qualitative claims of improvement. For forging-route comparison, the most relevant indicators include core effective strain, hydrostatic-compressive stress state, void-closure index, surface–core hardness difference, grain-size gradient, Charpy-impact retention, DBTT shift, and property scatter along the wall thickness. Classical free forging is advantageous in flexibility and equipment availability, whereas multi-axis deformation and radial-forging-type routes may improve strain-path diversity and circumferential uniformity when component size and tooling allow. Heat-treatment approaches should be compared using strength retention, impact-energy retention, carbide-coarsening index, retained-austenite decomposition degree, and surface–core property difference after tempering or PWHT. For this reason, each approach should be judged by a minimum evidence set consisting of one process parameter group, one internal-quality or microstructural metric, and one location-resolved mechanical-property or validation indicator.

The same logic applies to prediction methods. Empirical rules and forging-ratio criteria are transparent but weak in extrapolation; FEM-based process simulation provides stress/strain/temperature fields but requires reliable constitutive data and validation; surrogate and machine-learning models are efficient for plant-scale prediction but are vulnerable to data shift if alloy composition, ingot quality, or equipment conditions change; physics-informed models and digital twins offer the most promising route because they can combine reduced-order mechanics, transformation/precipitation models, sensor data, uncertainty quantification, and engineer-supervised updating. For industrial deployment, a digital twin should not be treated as a visualization tool alone, but as a closed-loop quality-control framework linking ingot records, pass schedule, real-time temperature/load/dimension data, heat treatment history, NDT results, and final mechanical-property feedback [37,41,42,43,48,49,69,74,75]. Prediction approaches should therefore report dataset scale, input-variable definitions, train/test or external-validation strategy, error metrics such as RMSE, MAE, or R2, and uncertainty or applicability limits when they are intended for industrial quality assurance.

To make the comparison less conceptual and more reproducible, Table 4 converts the process, heat-treatment, and prediction discussions into a standardized evidence matrix. The table separates the variables that should be reported, the quantitative indicators that allow cross-study comparison, and the interpretation limits that should be considered when transferring conclusions to super-thick 12Cr2Mo1V shell forgings.

This standardized matrix also clarifies the limits of the available evidence. Studies that report only final average strength or a nominal forging ratio are useful for qualitative discussion, but they are not fully comparable unless they also provide sampling position, local strain or void-closure evidence, microstructural descriptors, and validation error for any predictive model. This distinction helps prevent overgeneralization from individual case studies to industrial super-thick shell forgings.

### 6.4. Future Research Directions

Future research on 12Cr2Mo1V heavy-wall shell forgings should move beyond qualitative interpretation toward a quantitative and predictive metallurgical framework. A first priority is explicit modeling of through-thickness gradients in grain size, bainite morphology, carbide state, and hydrogen-related behavior. Recent studies on 12Cr2Mo1V large cylindrical forgings show that industrial production data can be combined with machine-learning models to predict grain size as well as room-temperature and high-temperature tensile properties, demonstrating that process–microstructure–property relationships in this alloy system can be quantified more effectively than by trial-and-error approaches alone [48,49].

**Figure 18:**
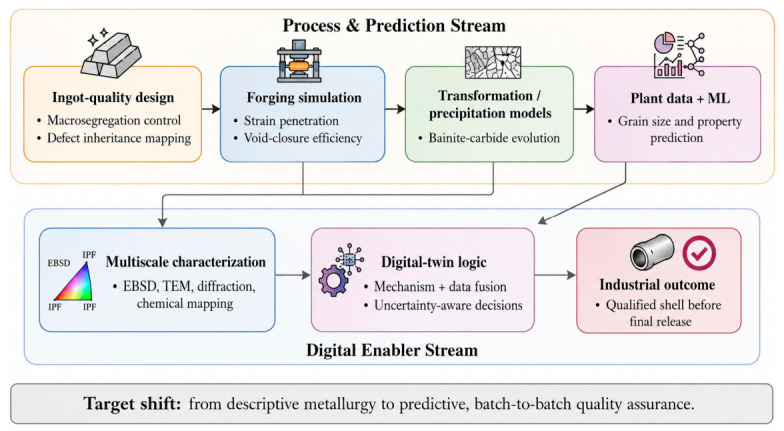
Roadmap for predictive quality control of 12Cr2Mo1V shell forgings through casting–free-forging–heat-treatment integration, multiscale characterization, digital-twin logic, and data-enhanced modeling.

A second priority is to deepen the study of process coupling across scales. For very large shell sections, the key scientific issue is no longer a single deformation or heat-treatment parameter, but the chain relationship among casting-derived heterogeneity, local strain penetration during forging, prior-austenite evolution, transformation behavior, carbide precipitation, and final through-thickness properties. Future research should therefore target integrated models that connect defect inheritance, deformation path, and heat-treatment response instead of optimizing these stages separately. As summarized in Figure 18, a forward-looking predictive-quality-control roadmap should include two interconnected streams: a process and prediction stream involving ingot-quality design, forging simulation, transformation/precipitation modeling, and plant-data-based machine learning; and a digital-enabler stream involving multiscale characterization, digital-twin logic, and uncertainty-aware decision support.

A third direction is the expansion of digitalization from property prediction to full process–structure–quality optimization. Although recent machine-learning work on 12Cr2Mo1V is encouraging because it identifies process variables that strongly influence grain size and mechanical performance, the next step should be more ambitious: data-driven models should be fused with physically informed constitutive, transformation, and precipitation models so that prediction is constrained by metallurgy rather than relying only on statistical correlation. Equally important is targeted multiscale characterization. Electron backscatter diffraction (EBSD), transmission electron microscopy (TEM), diffraction, and chemical mapping should be used not only to describe segregation bands, carbide chemistry, and bainitic heterogeneity, but also to quantify how these local features alter local mechanical response and hydrogen susceptibility. Ultimately, the most valuable future framework is likely to be a hybrid one in which ingot-quality information, free-forging simulation, transformation and precipitation modeling, multiscale characterization, and plant-scale production data are integrated to predict, before final heat treatment is completed, whether a shell will achieve qualified internal quality and acceptable through-thickness performance [39,48,49,69,74,75].

## 7. Conclusions

In summary, the performance of 12Cr2Mo1V hydrogenation reactor shells produced by free forging is governed by the coupled relationship among alloy design, inherited ingot quality, thermomechanical processing, microstructural evolution, and hydrogen-related service degradation. Three conclusions can be drawn from this review.

(1) 12Cr2Mo1V steel is metallurgically suitable for heavy-wall hydrogen-service shells because its Cr-Mo-V alloy design supports bainitic transformation, carbide stability, tempering resistance, and elevated-temperature performance. However, these advantages are realized only when forging and heat treatment jointly control bainitic morphology, carbide evolution, and hydrogen-trapping behavior across the full section thickness.
(2) Free forging is not only an appropriate forming route for large reactor shell forgings but also the decisive operation for defect closure, strain penetration, centerline consolidation, and precursor-structure formation. Nevertheless, segregation-related heterogeneity and thickness-dependent microstructural gradients remain persistent challenges in very large sections.
(3) Future progress should move from empirical process control toward predictive quality assurance. Integrated casting–free-forging–heat-treatment design, multiscale characterization, mechanism-based modeling, and data-driven tools should be combined to control through-thickness quality, reduce property scatter, and improve the reliability of 12Cr2Mo1V hydrogenation reactor shell forgings. In particular, future studies should establish standardized surface–core sampling schemes, quantitative bainite/carbide/retained-austenite descriptors, and digital-twin-assisted quality prediction so that different forging routes and heat-treatment strategies can be compared on a measurable basis. Comparable numerical indicators, including core effective strain, void-closure index, grain-size gradient, surface–core property scatter, DBTT shift, and model-validation error, should be reported consistently so that individual studies can be transformed into cumulative engineering evidence.

## Figures and Tables

**Table 1:** Condensed literature framework used to define the review scope and evidence chain.

Review Domain	Key Scope	Role in This Review
Service/alloy basis	Hydrogen-service requirements; 12Cr2Mo1V/12Cr2Mo1VR and related 2.25Cr-1Mo-0.25V steels	Defines the material-selection logic and reliability requirements of thick shell forgings.
Ingot defects and free forging	Macrosegregation, shrinkage porosity, inclusions, upsetting, cogging, drawing, and void closure	Links casting inheritance and deformation path with internal densification.
Bainite–carbide evolution	Granular/lath bainite, M/A constituents, retained austenite, carbide precipitation, tempering, and PWHT	Explains strength, toughness, tempering stability, and hydrogen trapping.
Through-thickness performance	Hardness, tensile properties, Charpy toughness/DBTT, hydrogen embrittlement, HTHA, fatigue, and creep	Connects microstructural gradients and defect state with section-wide service reliability.
Characterization and prediction	EBSD, TEM, chemical mapping, 3D reconstruction, FEM, machine learning, and digital-twin monitoring	Provides tools for quantitative comparison and predictive quality control.

**Table 2:** Simplified process-control framework for heavy-wall shell forgings.

Stage	Key Variables	Metallurgical Role	Main Risk If Uncontrolled
Ingot casting	Hot top design; filling rate; cooling path	Sets the initial segregation and shrinkage state	Centerline looseness and chemistry gradients
Breakdown/upsetting	Reduction per pass; die geometry; bite ratio	Promotes core strain penetration and hydrostatic compression	Incomplete internal void closure
Cogging/shell forming	Pass schedule; feed/rotation; temperature control	Maintains densification while forming the shell	Underworked core despite dimensional accuracy
Cooling/normalizing/quenching	Section size; cooling rate; thermal path	Establishes bainite morphology and surface–core gradients	Coarse granular bainite or unstable M/A constituents
Tempering/PWHT	Time–temperature history	Regulates carbide evolution and strength–toughness balance	Carbide coarsening or clustered precipitation
Service exposure	Hydrogen pressure; temperature; exposure time	Controls hydrogen diffusion, trapping, and HTHA susceptibility	Embrittlement or carbide destabilization

**Table 3:** Compact indicators for comparing through-thickness property uniformity in heavy Cr-Mo-V reactor-steel forgings.

Comparison Target	Recommended Indicators	Interpretation for Shell-Forging Quality
PWHT sensitivity	Hardness, Charpy energy, yield/tensile strength, carbide size, and bainite-lath coarsening	Thermal exposure can degrade toughness and strength through coarsening and carbide redistribution.
Surface–core heterogeneity	Location-resolved hardness, tensile/impact data, DBTT, bainite type, M/A morphology, and retained-austenite fraction	Surface data alone cannot represent core performance; central sampling is essential.
Defect/strain state	Local effective strain, hydrostatic compression, void-closure index, residual porosity, and segregation intensity	Mechanical scatter should be interpreted together with deformation and defect-closure maps.
Reporting standard	Sampling coordinates, heat-treatment history, mechanical data, and quantitative bainite-carbide descriptors	Enables comparable assessment of forging routes, heat treatments, and predictive models.

**Table 4:** Standardized comparative criteria for forging-route, heat-treatment, and prediction strategies relevant to 12Cr2Mo1V and related Cr-Mo-V heavy-wall reactor-steel forgings.

Approach or Technology	Minimum Variables and Numerical Metrics That Should Be Reported	Interpretation for Cross-Study Comparison
Conventional free forging/breakdown cogging	Forging ratio, reduction per pass, bite ratio, feed/rotation schedule, deformation temperature, local effective strain, hydrostatic stress, and void-closure index; useful anchors include a local effective strain of about 0.6 and ultrasonic confirmation of cavity closure near a forging ratio of 2.9S in reported large-ingot studies [51,52,53,54,55,56,57,58,59,60,61,62,63].	Provides the most direct evidence for densification and core working, but results remain strongly dependent on ingot size, die geometry, pass schedule, and initial defect morphology.
Multiaxial deformation	Number of loading directions, cumulative strain, temperature window, strain-path sequence, core effective-strain distribution, anisotropy index, and grain-size gradient.	Useful for comparing strain-path diversity and internal uniformity; transferability requires similar workpiece geometry and comparable thermal histories.
Radial forging/mandrel-assisted shell expansion	Radial reduction, feed per stroke, mandrel size, wall-thickness strain distribution, circumferential property scatter, NDT results, and dimensional deviation.	Can support circumferential uniformity and dimensional repeatability, but conclusions for ultra-large reactor shells must consider tooling rigidity, mandrel design, and size limits.
Normalizing, quenching, tempering, and PWHT	Austenitization or normalizing temperature/time, cooling rate, tempering or PWHT duration, hardness and yield/tensile retention, −30 °C impact energy, DBTT shift, retained-austenite decomposition, and carbide-coarsening index [7,16,17,18,19,29,33].	Allows comparison of heat-treatment sensitivity and surface–core property stability; data should be separated by sampling location rather than averaged over the section.
FEM/CAE process simulation	Constitutive equation, friction factor, heat-transfer coefficient, mesh sensitivity, predicted load, temperature field, core effective strain, hydrostatic stress, and void-volume or closure evolution validated by NDT or metallography [51,52,53,54,55,56,57,58,59,60,61,62,63,65,66,67,68].	Appropriate for comparing mechanism-based process feasibility, but reliability depends on boundary-condition calibration and experimental validation.
Surrogate and machine-learning prediction	Dataset size, production-batch coverage, input-variable definitions, feature importance, train/test split, external validation, RMSE, MAE, R2, and prediction intervals for grain size or mechanical properties [48,49,74,75].	Useful for plant-scale prediction and optimization, but vulnerable to data shift when alloy chemistry, ingot quality, press route, or heat-treatment practice changes.
Digital-twin-assisted quality control	Real-time load, stroke, temperature, geometry, NDT, heat-treatment, and mechanical-property feedback; model-update frequency, uncertainty bounds, false-accept/false-reject rate, and final qualification accuracy [37,41,42,43,69,74,75].	Most suitable for closed-loop quality assurance, but it requires traceable data architecture, uncertainty management, and engineer-supervised model updating.

## Data Availability

No new data were created or analyzed in this review. Data sharing is not applicable to this article.

## Abbreviations

The following abbreviations are used in this manuscript:

API	American Petroleum Institute
CAE	Computer-aided engineering
DBTT	Ductile-to-brittle transition temperature
DRX	Dynamic recrystallization
EBSD	Electron backscatter diffraction
FEM	Finite element method
HTHA	High-temperature hydrogen attack
M/A	Martensite-austenite constituent
PWHT	Post-weld heat treatment
TEM	Transmission electron microscopy
